# Efficacy of Transcranial Direct Current Stimulation (tDCS) on Neuropsychiatric Symptoms in Multiple Sclerosis (MS)—A Review and Insight into Possible Mechanisms of Action

**DOI:** 10.3390/jcm13247793

**Published:** 2024-12-20

**Authors:** James Chmiel, Marta Stępień-Słodkowska

**Affiliations:** 1 Faculty of Physical Culture and Health, Institute of Physical Culture Sciences, University of Szczecin, Al. Piastów 40B blok 6, 71-065 Szczecin, Poland; 2 Doctoral School of the University of Szczecin, University of Szczecin, Mickiewicza 16, 70-384 Szczecin, Poland

**Keywords:** tDCS, transcranial direct current stimulation, multiple sclerosis, non-invasive brain stimulation, neurostimulation, neuromodulation, depression, anxiety, transcranial electrical stimulation, neuropsychiatric symptoms

## Abstract

**Introduction**: Neuropsychiatric symptoms such as depression and anxiety are a significant burden on patients with multiple sclerosis (MS). Their pathophysiology is complex and yet to be fully understood. There is an urgent need for non-invasive treatments that directly target the brain and help patients with MS. One such possible treatment is transcranial direct current stimulation (tDCS), a popular and effective non-invasive brain stimulation technique. **Methods:** This mechanistic review explores the efficacy of tDCS in treating depression and anxiety in MS while focusing on the underlying mechanisms of action. Understanding these mechanisms is crucial, as neuropsychiatric symptoms in MS arise from complex neuroinflammatory and neurodegenerative processes. This review offers insights that may direct more focused and efficient therapeutic approaches by investigating the ways in which tDCS affects inflammation, brain plasticity, and neural connections. Searches were conducted using the PubMed/Medline, ResearchGate, Cochrane, and Google Scholar databases. **Results:** The literature search yielded 11 studies to be included in this review, with a total of 175 patients participating in the included studies. In most studies, tDCS did not significantly reduce depression or anxiety scores as the studied patients did not have elevated scores indicating depression and anxiety. In the few studies where the patients had scores indicating mild/moderate dysfunction, tDCS was more effective. The risk of bias in the included studies was assessed as moderate. Despite the null or near-null results, tDCS may still prove to be an effective treatment option for depression and anxiety in MS, because tDCS produces a neurobiological effect on the brain and nervous system. To facilitate further work, several possible mechanisms of action of tDCS have been reported, such as the modulation of the frontal–midline theta, reductions in neuroinflammation, the modulation of the HPA axis, and cerebral blood flow regulation. **Conclusions:** Although tDCS did not overall demonstrate positive effects in reducing depression and anxiety in the studied MS patients, the role of tDCS in this area should not be underestimated. Evidence from other studies indicates the effectiveness of tDCS in reducing depression and anxiety, but the studies included in this review did not include patients with sufficient depression or anxiety. Future studies are needed to confirm the effectiveness of tDCS in neuropsychiatric dysfunctions in MS.

## 1. Introduction

Neuropsychiatric symptoms such as anxiety and depression are prevalent in multiple sclerosis (MS). Depression affects approximately 30.5% of MS patients, while anxiety impacts around 22.1% [1,2]. Many patients experience both conditions concurrently [1]. These mood disorders have far-reaching consequences, including the exacerbation of MS symptoms [3], increased medication utilization [1], and inadequate disease management [4]. Additional consequences include decreased rates of employment [5], the reduced effectiveness of interventions [3], a diminished quality of life [6], and a heightened risk of suicidal thoughts and attempts [7]. Anxiety similarly reduces quality of life [8], increases suicidal ideation [9], and worsens medical adherence [10].

The effective treatment of these mood disorders is essential for improving quality of life, therapy adherence, and daily functioning in MS patients [11]. Current strategies often involve individualized plans combining pharmaceutical and non-pharmacological interventions [11]. Despite its prevalence, depression is frequently underdiagnosed or inadequately treated by neurologists [12]. Common treatments include antidepressant medications [13], cognitive behavioral therapy (CBT) [14,15], and physical exercise [16,17]. However, a universally accepted standard of care for depression in MS has not been established [18]. For anxiety, the evidence base is even more limited [19]. Preliminary findings suggest that CBT [15] and self-management interventions [20] may hold promise for addressing anxiety in this population.

MS is associated with specific neural damage, such as demyelination, grey and white matter atrophy, and network disruptions, which significantly contribute to the development of depression and anxiety [21]. Demyelination and axonal damage impair neural transmission, particularly affecting brain networks like the default mode network (DMN) [22], which is crucial for self-referential thoughts and emotional regulation [23]. This disruption is compounded by damage to white matter, which disconnects key cortical and subcortical regions involved in emotional processing and stress management [21]. Grey matter atrophy is strongly associated with mood regulation impairments, particularly in regions like the dorsolateral prefrontal cortex (DLPFC), anterior cingulate cortex (ACC), hippocampus, and amygdala [21]. The DLPFC, essential for cognitive control [24], often exhibits functional decline in MS patients with depression, reducing their ability to manage stressors effectively [21]. The hippocampus and amygdala, integral to memory [25] and emotional processing (ref. [26]) are also adversely affected. Atrophy in these regions not only impairs memory but also increases the vulnerability to anxiety by heightening the sensitivity to stressors and negative emotional stimuli [21].

As there is no established effective treatment for neuropsychiatric disorders in MS [21], there is an urgent need to develop safe new non-invasive treatments. Depression and anxiety in MS involve complex pathophysiological mechanisms in the brain [21], so ideal treatments would be those that directly target the brain. One such treatment may be transcranial direct current stimulation (tDCS), a non-invasive brain stimulation technique that has been gaining much clinical and neuroscientific interest in recent years.

The fundamental tenet of tDCS is that a small electrical current can alter plasticity in the brain during ongoing brain activity, potentially leading to behavioral changes [27]. Using two or more electrodes, tDCS applies a constant low-intensity electrical current (about 2 mA) to the target brain area beneath the electrodes, as well as potentially influencing distant brain regions through network connectivity [28]. Anodal stimulation often increases excitability in the area under the anode and cathodal stimulation decreases excitability, where the direction of polarization is solely dependent on the orientation of the axons and dendrites of the brain region targeted by the electrical stimulation [29]. When a neuron receives input from another, this current modifies the likelihood that the neuron will fire at a subthreshold level [30]. Transcranial magnetic stimulation (TMS), another neurostimulation technique often used to measure cortical excitability, has shown that anodal tDCS enhances excitability over the primary motor cortex (M1), while cathodal tDCS decreases it [31]. Apart from the immediate impacts, tDCS has the ability to cause enduring modifications at the synaptic level via mechanisms akin to long-term potentiation (LTP) and depression (LTD), respectively, for anodal and cathodal stimulation [32]. This results in an improvement in neuroplasticity [32]. Based on a number of pharmacological investigations, it is believed that the neuroplastic mechanism of action of tDCS is due to the calcium-dependent synaptic plasticity of glutamatergic neurons, as tDCS effects are lessened when N-methyl d-aspartate (NMDA) receptors are blocked [32]. Regardless of the anodal or cathodal stimulation polarity, tDCS can locally decrease gamma-aminobutyric acid (GABA) neurotransmission in addition to excitatory activity [32]. tDCS has been shown to be effective in reducing the symptoms of many conditions, such as ADHD [33], autism [33], dyslexia [33], migraine [34], and many others.

There are several reviews of studies that have examined the effectiveness of tDCS in improving pain, fatigue, gait, balance, and cognitive functions in MS patients [35,36,37,38,39]. In addition, many studies have demonstrated the effectiveness of tDCS in treating depression [40] and anxiety [41]. A 2024 meta-analysis also showed that tDCS can be an effective tool in treating depression and anxiety that occur as comorbid disorders in another condition [40]. However, no work has investigated the effect of tDCS on neuropsychiatric symptoms in MS. The aim of this review will be to fill this gap and investigate the effect of tDCS on symptoms of anxiety and depression in patients with MS. This review specifically adopts a mechanistic approach, which focuses on understanding how tDCS exerts its effects on neuropsychiatric symptoms in MS, such as depression and anxiety. Mechanistic reviews are essential for elucidating the biological and physiological pathways through which interventions produce therapeutic effects. Given the complex interplay of neuroinflammation, demyelination, and network disruptions in MS, understanding the mechanisms of tDCS (e.g., the modulation of neural oscillations, synaptic plasticity, and inflammatory responses) can help improve intervention protocols, tailor treatments to patient needs, and facilitate future clinical trials. By identifying causal pathways, this review aims to bridge the gap between observed clinical effects and their neurobiological underpinnings, advancing the use of tDCS as a non-invasive therapeutic option for MS-related mood disorders.

## 2. Methods

This mechanistic review aims to systematically evaluate the efficacy of tDCS in alleviating neuropsychiatric symptoms, specifically depression and anxiety, in MS patients. To ensure the reliability and relevance of the evidence included, a comprehensive literature search and rigorous selection criteria were employed. The methodology was guided by established practices for conducting systematic reviews and evidence synthesis (PRISMA) [42], with a focus on identifying clinical trials and case studies that assess the therapeutic impact of tDCS on MS-related neuropsychiatric dysfunctions. It is important to note that this is a mechanistic review rather than a systematic one, and it does not adhere to all elements of the PRISMA methodology typically used in systematic reviews.

### 2.1. Data Sources and Search Strategy

While preparing this review, J.Ch. and M.S.-S. used the following set of combined keywords to conduct an independent standards-abiding Internet search: “tDCS” OR “transcranial direct current stimulation” AND “multiple sclerosis”. We conducted a thorough search in September 2024 using several databases, including PubMed/Medline, Research Gate, Google Scholar, and Cochrane, with an emphasis on those published between January 2008 and September 2024.

### 2.2. Study Selection Criteria

To be eligible for this study, the publications had to be published in English between 2008 and 2024 and be clinical trials or case studies. This study had to look into how tDCS affected depression or anxiety in MS patients, either as a primary or secondary finding. All review papers and those that were not written in English were excluded.

### 2.3. Screening Process

Numerous screening processes were implemented to guarantee that pertinent research was included and that any studies that did not match the predetermined criteria were disregarded. Two independent reviewers, J.Ch. and M.S.-S., thoroughly reviewed the abstracts and titles during the first screening stage.

#### 2.3.1. Title and Abstract Screening

The titles and abstracts of the records that were independently accessible were assessed by each reviewer to ascertain which studies met the inclusion requirements. The impact of tDCS on depression and anxiety in MS patients was the main focus of the screening criteria.

#### 2.3.2. Full-Text Assessment

Following an initial screening of titles and abstracts, the selected papers underwent a comprehensive full-text evaluation. The reviewers carefully checked each publication to make sure it matched the eligibility conditions, paying special attention to whether the studies were published in English between January 2008 and September 2024 and were clinical trials or case studies.

## 3. Results

**Figure 1:**
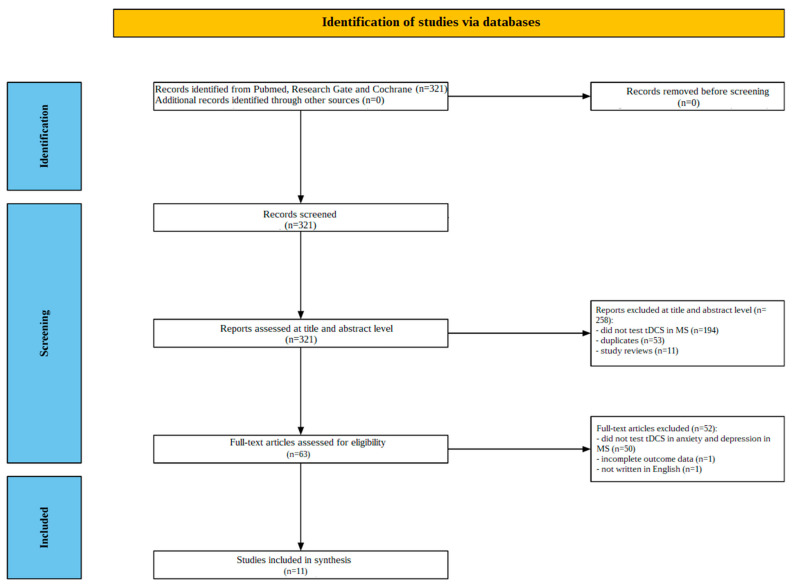
Flowchart depicting the different phases of the systematic review.

Figure 1 provides an illustration of the screening process. Initially, 321 studies were found in the numerous database search results; 258 articles were discarded after their titles and abstracts were reviewed, where 194 of these studies did not test tDCS in MS, 53 were duplicates, and 11 were research reviews. A comprehensive full-text analysis was carried out on the remaining 63 papers; 50 studies were then excluded because they did not investigate the effect of tDCS on depression or anxiety in MS patients, 1 was excluded because it contained incomplete outcome data, and 1 was excluded because it was written in a language other than English. An extensive analysis of the contents led to the determination that eleven (11) articles met the criterion for admission.

The eleven studies [43,44,45,46,47,48,49,50,51,52,53] were published between 2010 and 2024. Studies [43,46,48,51,53] were RCTs, [44] was a comparative study, [45] was an exploratory study, [47] was a prospective study, [49,52] were pilot studies, and [50] was a case study. Eight studies [43,44,45,46,47,49,51,53] were double-blind, one [48] was single-blind, and one [52] was not blinded.

This review employed four psychometric scales to assess neuropsychiatric symptoms: the Hospital Anxiety and Depression Scale (HADS), the Beck Depression Inventory (BDI), the Depression, Anxiety, and Stress Scale (DASS), and the Hamilton Depression Rating Scale (HDRS)**.** These tools are widely used in both clinical and research settings to evaluate anxiety, depression, and related emotional states.

The Hospital Anxiety and Depression Scale (HADS) is a 14-item scale designed specifically for medical populations to assess anxiety and depression while minimizing the influence of physical health symptoms. It comprises two subscales—anxiety and depression—each containing seven items. Responses are scored from 0 to 3, resulting in subscale scores ranging from 0 to 21. Scores are interpreted as follows: 0–7 indicates normal levels, 8–10 suggests borderline abnormal symptoms, and 11–21 reflects abnormal levels of anxiety or depression [54].

The Beck Depression Inventory (BDI) is a widely used 21-item scale that measures the severity of depressive symptoms. Each item is scored from 0 to 3, based on symptom intensity, yielding a total score ranging from 0 to 63. Scores are categorized as follows: 0–13 for minimal depression, 14–19 for mild depression, 20–28 for moderate depression, and 29–63 for severe depression. The BDI assesses various dimensions of depression, including emotional, cognitive, and physical symptoms [55].

The Depression, Anxiety, and Stress Scale (DASS) is a comprehensive tool that measures three distinct dimensions: depression, anxiety, and stress. It consists of 42 items, divided evenly across the three subscales. Subscale scores range from 0 to 42, with the following cut-offs for depression: 0–9 (normal), 10–13 (mild), 14–20 (moderate), 21–27 (severe), and 28+ (extremely severe). Similar cut-off scores apply to the anxiety and stress subscales, allowing for a detailed understanding of emotional distress [56].

The Hamilton Depression Rating Scale (HDRS), also known as the Hamilton Rating Scale for Depression (HAM-D), is a clinician-administered tool that evaluates the severity of depression. The HDRS typically consists of 17 items, although extended versions with up to 21 items exist. Items are scored on a scale of 0–2 or 0–4, with higher scores reflecting greater severity. The total score ranges from 0–52 for the 17-item version, with scores interpreted as follows: 0–7 (normal), 8–16 (mild depression), 17–23 (moderate depression), and 24+ (severe depression). The HDRS is widely regarded as the gold standard for assessing depressive symptoms in clinical research and requires proper training to ensure consistent administration and scoring [57].

### 3.1. Summary of Included Studies

The included studies are summarized in Table 1, the characteristics of the patients included in the studies are described in Table 2, and the assessment of the risk of bias in the included studies is presented in Table 3. Eleven MS patients (with a mean age of 43.91 years) participated in study [43] and were randomly assigned to undergo two blocks (active and sham tDCS) of five daily bifrontal tDCS sessions (anode/cathode over the left/right DLPFC, F3, F4) in a crossover fashion. The blocks were separated by a washout interval of three weeks. Assessments were conducted on day 1, day 5, immediately following each block, and one week later. In the 20 min sessions, tDCS was administered at a 2 mA intensity. The HADS was used to measure depression and anxiety. No numerical data were provided for HADS depression scores or anxiety scores before treatment. From the graph, it can be seen that the average HADS depression score was approximately 7 (no depression). From the graph, it can be seen that the average HADS anxiety score was approximately 5.6 (no anxiety).

In study [44], ten MS patients (with a mean age of 40.5 years) were included in a crossover, double-blind, sham-controlled trial. Three anodal tDCS treatment blocks were given to each subject at random: sham stimulation over either cortical region, active stimulation over the right posterior parietal cortex (PPC), and active stimulation over the left DLPFC (F3). The cathode was positioned above the right supraorbital area for the DLPFC stimulation. The appropriate anode was positioned over P4 and the matching cathode over Cz in order to stimulate the proper PPC. The current intensity was 2 mA and the stimulation lasted for 20 min. The blocks comprised five consecutive daily sessions and were separated by a three-week washout interval. The HADS depression score was used to measure depression. The mean HADS depression score before treatment in the DLPFC group was 3.9 and the score in the PPC group was 4.2. The results in both groups did not indicate the presence of depression. Anxiety was measured using the HADS anxiety score. The pre-treatment score was 5.7 in the PPC group and 4.6 in the DLPFC group (no anxiety).

In study [45], tDCS was administered over the left DLPFC (F3) of 13 MS patients (with a mean age of 46.8 years) for five days in a sham-controlled, double-blind intervention research. On the opposite forehead, the cathode was positioned. A month’s worth of symptoms were monitored through questionnaires. All patients received two blocks using a crossover design, one with sham stimulation and the other with actual stimulation in a pseudo-randomized counter-balanced manner. The stimulation lasted 20 min and the current intensity was 1 mA. The BDI and HADS depression scores were used to measure depression. Before treatment, the BDI score was 4 and the HADS score was 2. Both scores did not indicate depression. Anxiety was measured using the HADS anxiety score. The pre-treatment score was 4 (no anxiety).

In a randomized double-blind sham-controlled study [46], 19 MS patients (with a mean age of 44.8 years) were randomly assigned to receive real tDCS (*n* = 10) or sham tDCS (*n* = 9). Assessments were carried out at baseline, right after treatment concluded, and once a week for the next three weeks. The painful somatic area was contralateral to the anode electrode, which was positioned over the C3 or C4 location. A continuous current of 2 mA intensity was administered for 20 min once a day for 5 consecutive days. The supraorbital region, which is contralateral to the motor cortex that was activated, was covered by the cathode electrode. BDI was used to measure depression. No figures were given for the pre-treatment depression scores, but the graph shows that the average BDI score was approximately 11 (no depression). The VAS was used to measure anxiety. No numerical data were given, but, from the graph, it can be estimated that the score before treatment was around 40 (moderate anxiety).

An investigation [47] that was crossover, sham-controlled, and randomized involved sixteen MS patients (with a mean age of 48.9 years). Two anodal tDCS blocks—either sham or active—each lasting three weeks and comprising three straight daily tDCS sessions, were administered at random to the patients. Each block was evaluated both before and after. The left DLPFC was stimulated by positioning the anode over F3 and the matching cathode over the right supraorbital area. The current was 2 mA. The stimulation lasted 20 min. The HADS depression score was used to measure depression. The mean HADS score before treatment was 6.4, indicating no depression. The HADS anxiety score was used to measure anxiety. The score before treatment was 7.7 (normal–mild anxiety).

In study [48], five days of tDCS with an interval period was the subject of a novel protocol in a randomized controlled single-blinded trial. Thirty individuals in all were enrolled in the trial; fifteen were randomized to an active group (with a mean age of 51.2 years), while the remaining fifteen were assigned to a sham group (with a mean age of 49.87 years). Following a five-day a-tDCS course, pre-treatment and posttreatment outcomes were assessed every week for a maximum of four weeks. The C3 or C4 position, which is opposite the side of pain, was where the anodal electrode was placed. The supraorbital region, which is contralateral to the motor cortex that was activated, was covered by the cathode electrode. For five days in a row, at around the same time every day, a steady current of 2 mA intensity was delivered along with the following protocol: 10 min of stimulation, 25 min of nonstimulation, and then a further 10 min of stimulation. Depression and anxiety were measured using the DASS. The DASS depression subscale score before treatment in the active group was 6.9. This score indicated mild depression. The DASS anxiety subscale score before treatment in the active group was 7.9 (normal–mild anxiety).

In study [49], a double-blind, sham-controlled, randomized crossover pilot investigation was carried out by Workman et al. Six MS patients (with an average age of 46.7 years) undertook two randomly assigned stimulation blocks (tDCS or sham). There were five daily sessions in each block. Participants filled out the BDI questionnaire at both the initial visit and the end of treatment. For twenty minutes, the subjects were stimulated with 2 mA. The contralateral supraorbital region was covered by the cathode and the M1 was covered by the anode. The pre-treatment BDI score was 11.7, indicating no depression.

In case study [50], after multiple rounds of both unilateral and bilateral ECT treatments, Clayton et al. reported the extended tDCS therapies in an MS patient, a 54-year-old lady, with bipolar I illness and a 20-year history of severe recurring episodes of depression, in addition to a secondary progressive subtype of MS with significant neurological disability. The patient underwent 40 sessions of tDCS of the left DLPFC, with a current of 2 mA for 20 min. Depression was measured using the HDRS. The HDRS score before treatment was 15 (mild depression).

In the randomized, double-blind, sham-controlled study [51], 10 patients participated in the active tDCS group, and 10 in the sham tDCS group. Anodal stimulation was performed on the somatosensory cortex. The anode was positioned on the scalp, 2 cm posterior to the C3 or C4 position in order to target this area of the cortex. The supraorbital region, which is opposite to the activated sensory cortex, was covered by the cathode electrode. Five stimulations were provided for five days in a row, each lasting 20 min. A current of 2 mA was used. The BDI was used to measure depression. No numerical data were given for the BDI score before treatment, but, from the graph, it can be estimated that the average BDI score was about 12 (minimal depression).

Twelve participants took part in the crossover design trial [52]. The study involved 10 sessions of tDCS, followed by a concurrent exercise program, each lasting 4 weeks, with a 5-month washout period between interventions. There was no control group and no randomization. The anode was applied to the left DLPFC, and the cathode to the right supraorbital area. Sessions lasted 20 min over a period of 4 weeks. The current intensity was 2 mA. Depression was measured using the BDI. The pre-treatment score was 13.83 (minimal–mild depression).

In the randomized double-blind study [53], 37 patients participated. Using the block randomization method, the patients were assigned to two groups: one with sham stimulation (*n* = 20) and the other with 1.5 mA stimulation (*n* = 20) of the left dorsolateral prefrontal cortex (F3) and the right frontopolar cortex (Fp2), respectively, using anodal and cathodal stimulation. Ten 20 min stimulation sessions each week were part of the treatment. The DASS scale was used to measure anxiety and depression. The DASS depression score before treatment was 3.73, and the DASS anxiety score before treatment was 3 (no depression and anxiety).

### 3.2. Effects on Depression

In study [43], no significant differences in HADS depression scores were observed following active or sham interventions (Friedman’s test; X^2^ = 5.62; *p* = 0.345). No numerical data were provided regarding the outcomes after treatment. From the graph, it can be estimated that the HADS scores after treatment were about 6.

Study [44] showed that only the right PPC group showed a significant decrease in depression scores (from 4.2 to 1.9, no depression). The DLPFC group also showed a decrease, but it was not significant (from 3.9 to 2.3).

In study [45], there was no significant decrease in depression in HADS or BDI measurements (lack of numerical data).

In study [46], the BDI scores dropped, but the difference was not significant. The score in the fourth week, according to the graph, was about 7.

Study [47] showed that the HADS score dropped from 6.4 to 6.0, but this was not significant.

In study [48], the DASS score dropped from 6.9 to 6.6, but this was not significant (no depression).

In study [49], the BDI score dropped from 11.7 to 11.5, but this was not significant (minimal depression).

In case study [50], the HDRS score dropped from 15 to 11 (mild depression).

In study [51], tDCS did not improve depression scores in BDI (lack of numerical data, ~12 from the graph).

In study [52], a significant improvement in BDI was noted from 13.83 (minimal–mild depression) to 10.58 (minimal depression).

In study [53], tDCS reduced the DASS depression score from 3.73 to 2.06 (no depression).

### 3.3. Effects on Anxiety

In study [43], tDCS significantly reduced the HADS anxiety scores 1 week after the stimulation block. The score was not given, but, from the graph, it can be estimated that the score was around 3.6.

Study [44] showed that only the PPC group tDCS significantly reduced anxiety scores (from 5.7 to 2.0). The improvement in the DLPFC group was not significant (from 4.6 to 2.8).

In study [45], there was no significant decrease in anxiety in HADS (lack of numerical data).

In study [46], tDCS non-significantly reduced the VAS anxiety scores.

In study [47], tDCS non-significantly reduced the anxiety scores (from 7.7 to 7.6).

In study [48], the results showed that tDCS non-significantly reduced the anxiety scores (from 7.9 to 7.1).

In study [53], tDCS reduced the DASS anxiety score (from 3.00 to 1.60, no anxiety).

### 3.4. Bias Risk Assessment

Most studies employed adequate randomization methods, such as computer-based randomization, except for the case study and non-randomized trial. Allocation concealment was adequately maintained in most randomized trials, although it remained unclear in one study. The majority of studies ensured the double-blinding of participants and personnel, while one study was single-blind, and non-randomized studies lacked blinding altogether. Incomplete outcome data presented a low risk of bias across all studies, as most participants completed the protocols without significant attrition. However, selective reporting was a concern, as several studies presented results graphically instead of numerically, increasing the risk of reporting bias. Overall, most studies exhibited a moderate risk of bias, primarily due to incomplete or unclear reporting, while the case study and non-randomized studies carried a high risk of bias.

## 4. Discussion

Neuropsychiatric symptoms in MS, such as depression and anxiety, are an additional burden for patients. Their pathophysiology is complex and not yet fully understood. It is known that neuropsychiatric dysfunctions in MS manifest through changes in the nervous system, including the brain. It is therefore important to develop interventions that target the brain. This review examines the use of transcranial direct current stimulation, a non-invasive brain stimulation technique, in treating symptoms of depression and anxiety in MS. The findings indicate that the effectiveness of tDCS in addressing neuropsychiatric symptoms in MS remains inconclusive. Most of the included studies reported non-significant changes in depression and anxiety scores, likely due to the absence of pre-treatment symptoms in participants. However, studies involving patients with mild-to-moderate baseline symptoms showed some potential for tDCS to improve outcomes. This review not only evaluates these findings but also examines potential mechanisms of action, providing insights for future research and clinical applications (discussed in Section 5).

The reviewed studies on the effects of tDCS on depression in MS patients present a varied picture, with the majority reporting non-significant changes in depression scores. In most studies, MS patients are not depressed in pre-intervention measures, and it is difficult to treat a condition that is not there. To be more specific, in study [43], there were no significant differences in the HADS depression scores after the active or sham stimulation, with the average post-treatment score estimated at around 6. Similarly, study [45] found no significant changes in depression based on the HADS and BDI scores. Study [46] reported a drop in BDI scores from approximately 11 to 7, but this was not significant. Studies [47,48] demonstrated small reductions in the HADS and DASS depression scores, although neither was significant. In contrast, studies [44,52,53] showed more promising results. Study [44] reported a significant decrease in depression scores (from 4.2 to 1.9) in the right PPC group, although the DLPFC group did not show significant improvements. Study [52] showed that tDCS applied to the left DLPFC works in the case of depression, even mild depression. Furthermore, this study used a larger number of sessions than the other studies—10 stimulation sessions over a 4-week period, not just 5 sessions. These results are consistent with the findings that the treatment of depression with tDCS must include a minimum of 10 sessions, and that the stimulation of the left DLPFC is an effective protocol. Study [53] noted a reduction in the DASS depression score from 3.73 to 2.06. Additionally, case study [50] showed a marked reduction in HDRS depression scores from 15 to 11 following extended tDCS sessions.

A notable limitation in interpreting tDCS results for depression in MS is the absence of numerical data in some studies. For instance, in studies such as [43], outcomes are presented graphically, making it impossible to calculate effect sizes or perform a meta-analysis. As a result, the findings for this review had to be estimated visually.

Although the improvement in depression in the included studies was non-significant, tDCS may still be a promising intervention in reducing depression symptoms in patients with MS, but only statistically significantly if the patients actually exhibit severe depression symptoms. In patients without these problems, stimulation will not have any measurable effect. This is consistent with the results of reviews where tDCS reduces depression symptoms [58,59,60]. There is ample evidence that the stimulation of the left DLPFC is effective. It has also been shown that the effects of the treatment are long-term [59]. Furthermore, tDCS is an effective adjunctive treatment option to other interventions, such as medication or psychotherapy. Since there is no established treatment that has been proven effective for depression in MS, such an adjunctive treatment in the form of tDCS may be particularly effective, considering its low cost and safety.

The impact of tDCS on anxiety in MS patients also presents mixed outcomes, with some studies demonstrating significant reductions in anxiety scores, while others report non-significant changes. Once more, in most studies, patients did not provide pre-treatment measures that would indicate the presence of an anxiety disorder. In study [43], tDCS significantly reduced HADS anxiety scores one week post-treatment, with the score estimated to decrease from 5.6 to around 3.6. Similarly, study [44] observed a significant reduction in anxiety in the right PPC group, where scores dropped from 5.7 to 2.0, although improvements in the DLPFC group were non-significant (4.6 to 2.8). In contrast, study [45] reported no significant changes in anxiety scores post-tDCS, and study [46] noted a non-significant reduction in VAS anxiety scores. Studies [47,48] also observed minimal non-significant reductions in anxiety, with scores decreasing from 7.7 to 7.6 and 7.9 to 7.1, respectively. Study [53] found a substantial decrease in DASS anxiety scores with a significant drop from 3.00 to 1.60. These results suggest that tDCS may have a positive effect on anxiety in some MS patients, particularly when stimulating specific brain regions like the PPC.

As with depression, some studies examining the effects on anxiety did not provide complete numerical data on anxiety outcomes. This makes the analysis and interpretation of results difficult and precludes the conduct of a meta-analysis.

As with depression, tDCS for anxiety in MS patients may still be a promising intervention. Review papers have shown that tDCS alleviates the symptoms of anxiety [40,41]. One effective protocol is the stimulation of the left DLPFC, the same brain region involved in depression. Depression and anxiety in MS often occur together, so, by using tDCS to the left DLPFC, we can target both of these dysfunctions. Furthermore, research suggests that tDCS for anxiety disorders is an excellent complementary treatment option to psychotherapy or pharmacotherapy.

Previous research has established tDCS as a non-invasive technique capable of modulating brain plasticity and improving symptoms of depression and anxiety. This study builds upon that body of work by focusing on a population with unique challenges—MS patients, whose neuropsychiatric symptoms arise from complex interactions of neuroinflammation, neurodegeneration, and disrupted brain networks. Although the current evidence does not strongly support the efficacy of tDCS in MS, the findings align with prior studies that highlight the importance of tailoring stimulation protocols to individual patient characteristics, such as the baseline symptom severity.

These results have several implications. First, they underscore the importance of including patients with clinically significant baseline symptoms in future research. Neuropsychiatric disorders in MS are often underdiagnosed, and interventions like tDCS may have limited observable effects in patients without measurable pre-treatment dysfunctions. Second, the findings highlight the need to standardize stimulation parameters, such as the session duration, intensity, and frequency, to optimize therapeutic outcomes. The reviewed studies also suggest that targeting specific brain regions, such as the left dorsolateral prefrontal cortex, may be more effective in alleviating both depression and anxiety symptoms.

This review contributes to advancing the field by identifying key challenges and opportunities for future research. The ultimate goal is to establish tDCS as a reliable, non-invasive treatment for neuropsychiatric symptoms in MS. Achieving this requires addressing several challenges, including small sample sizes, methodological inconsistencies, and limited long-term follow-up data. Additionally, further studies are needed to elucidate the mechanisms through which tDCS exerts its effects, such as modulating neuroinflammation, enhancing cerebral blood flow, and regulating brain oscillatory activity (discussed later).

This review, the first of its kind on this topic, contributes to the growing interest in non-invasive brain stimulation methods, particularly tDCS, for the treatment of MS. The large number of studies examining the effectiveness of tDCS in MS (63 identified in the literature search for this paper) underscores the significant interest in this topic within the field of brain stimulation research. Previous reviews have examined the effectiveness of tDCS in MS in reducing pain or fatigue. These symptoms are also a significant burden for patients, but the effect of tDCS on the neuropsychiatric condition in this group of patients has yet to be analyzed. This review aims to fill the gap in knowledge.

It can be assumed that interest in the use of tDCS in MS will continue to grow. This is primarily due to the increasing prevalence of MS in the general population [61]. The growing number of MS patients is prompting mainstream medicine to seek effective and safe treatments. As previously discussed, there is no gold standard for the treatment of neuropsychiatric disorders in MS. While this review does not demonstrate the effectiveness of tDCS in this regard, it highlights a significant gap in knowledge and research that needs to be addressed. There is cautious optimism, based on the limited data presented in this review, that tDCS may benefit MS patients with significant anxiety and depression. As the number of people with MS increases, so does the prevalence of emotional dysfunctions in this population. Although the research synthesis in this paper yielded negligible results, it highlights areas of MS research that warrant further exploration.

Despite its merits, this review is not without limitations. The small number of studies, heterogeneous methodologies, and reliance on graphical rather than numerical data in some cases limit the generalizability of the findings. Additionally, the absence of meta-analytic approaches due to data variability precludes robust statistical conclusions.

## 5. Potential Mechanisms of Action of tDCS in Alleviating Depressive and Anxiety Symptoms in MS

**Figure 2:**
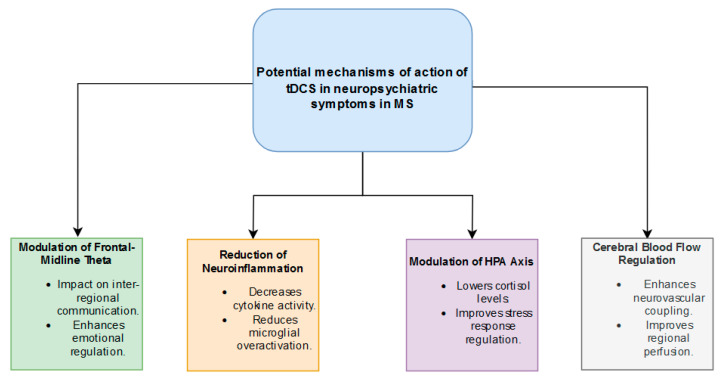
Flowchart describing potential mechanisms of action of tDCS in depressive and anxiety symptoms in MS.

The therapeutic effects of tDCS in reducing symptoms of depression and anxiety in MS are complex and as yet unexplored. It is worth discussing the possible mechanisms of tDCS action to facilitate future research in this area. It should be emphasized, however, that these hypothetical mechanisms are speculative in nature and need to be verified in studies on patients diagnosed with depression and anxiety in MS. A graphical representation of the mechanisms of tDCS action is presented in Figure 2.

### 5.1. Modulation of Frontal–Midline Theta

In study [47], tDCS increased frontal–midline theta oscillations in MS patients, where it may be linked to several underlying mechanisms involving key brain regions associated with anxiety regulation, such as the hippocampus [62], prefrontal cortex (PFC) [63], and amygdala [64]. Theta oscillations, especially in the hippocampus and PFC, are crucial for inter-regional communication and synchronization, particularly in contexts related to anxiety and fear [65]. As their appearance seems to be closely related to improved anxiety symptoms under treatment with anti-anxiety drugs, frontal–midline theta oscillations have been suggested as an instrument to evaluate symptoms of anxiety in generalized anxiety disorder [66]. The first potential mechanism is that tDCS might increase these oscillations by modulating the ventral hippocampal (vHPC) projections to the medial prefrontal cortex (mPFC), which have been shown to synchronize during anxiety-like behaviors [67]. This synchronization, especially in the theta range, facilitates communication between the vHPC and mPFC, crucial for encoding and processing anxiogenic stimuli [65]. Furthermore, the PFC’s role in the top-down regulation of emotional responses, including fear and anxiety, suggests that a tDCS-induced increase in theta oscillations may enhance PFC control over subcortical structures like the amygdala, known to drive anxiety-related behavior through its connection with the hippocampus. Reinforcing these oscillatory networks by tDCS may reduce hyperactivity in anxiety-related circuits, promoting an anxiolytic effect. Another potential mechanism is the reinforcement of local neural networks through increased excitability, which may help realign dysfunctional oscillatory patterns contributing to anxiety in MS patients. tDCS, by influencing these theta rhythms, likely enhances the brain’s ability to regulate fear and stress responses, which, in turn, leads to improvements in anxiety scores.

There is a strong correlation between the frontal–midline theta and activity in the brain regions that make up the “default mode network” (DMN) [68], which is closely linked to the mPFC [69]. In the anterior cingulate cortex, the mPFC, precuneus, posterior cingulate, and angular gyrus are all regions that are part of the DMN—for example, Scheeringa et al. [70] demonstrated a negative correlation between the frontal–midline theta power and blood-oxygenation-level-dependent (BOLD) signal. These data were obtained while the subjects were at rest. There is ample evidence that the DMN is hyperactivated in depression [71], which may be reflected in the lower frontal–midline theta power. tDCS, which increases the frontal–midline theta, may reduce the DMN activity, leading to a reduction in depressive symptoms in MS.

### 5.2. Reduction of Neuroinflammation

Neuroinflammation is a central feature in both MS and depression, with cytokines playing a critical role in the pathogenesis of both conditions [72]. In MS, the breakdown of the blood–brain barrier allows immune cells to infiltrate the central nervous system (CNS), leading to demyelination and axonal damage [73]. These immune dysregulations, characterized by elevated levels of proinflammatory cytokines such as IL-1β, IL-6, and TNF-α, are associated with both MS progression and depressive symptoms [21]. Similar to MS, depression has been linked to increased cytokine levels in peripheral blood, suggesting that immune system activation could contribute to mood disturbances [74]. Animal models of MS have demonstrated that behavioral changes, such as anhedonia and decreased social interaction, occur alongside the increased expression of proinflammatory cytokines in the brain [75]. These findings support the idea that inflammation-mediated neurodegeneration and immune cell activation are key contributors to depression in MS. Cytokines such as IL-1β, TNF-α, and IFN-γ have been shown to disrupt neuroendocrine function by activating the hypothalamic–pituitary–adrenal (HPA) axis, resulting in hypercortisolemia and glucocorticoid resistance [76]. This leads to the depletion of neurotransmitters like serotonin and norepinephrine, both of which are crucial for mood regulation [77]. In MS, neuroinflammation not only damages myelin but also alters synaptic function, potentially contributing to depressive symptoms [75]. Furthermore, the involvement of immune cells such as T cells and microglia in MS pathology exacerbates neurodegenerative processes, creating a feedback loop that worsens both MS and depression [78].

The role of neuroinflammation in anxiety disorders in MS is poorly understood. Limited evidence suggests that high levels of pro-inflammatory markers, such as IL-2, correlate with high scores on anxiety tests in MS patients [79]. It has been demonstrated that cytokines significantly impact glutamate transmission [80]. IL-2, which has been linked to anxiety in this instance, affects the way the brain’s HPA axis functions, as well as how different neurotransmitters are modulated [81].

tDCS applied to the left DLPFC in patients with depression has been shown to reduce markers of neuroinflammation [82,83]. One potential mechanism involves the reduction in the secretion of cytokines by tDCS, which activate the HPA axis and disrupt the serotonin and norepinephrine signaling pathways, crucial for mood regulation. Decreasing these cytokines may lead to improvements in both depressive and anxiety symptoms. Additionally, tDCS may attenuate microglial activation in the brain. Microglia, when overactivated, produce excessive amounts of pro-inflammatory cytokines and contribute to neurodegeneration. This neuroinflammatory state in MS, particularly in progressive forms, has been associated with synaptic alterations and neurodegenerative markers. Reducing microglial activity by tDCS might decrease the inflammatory response and protect against further neurodegeneration, providing neuroprotective effects that mitigate both MS progression and depressive symptoms.

### 5.3. Modulation of HPA Axis

The principal system of the body to respond to stress is the hypothalamic–pituitary–adrenal (HPA) axis. There is ample evidence that the HPA axis is dysregulated in MS, with some patient subsets exhibiting both hyperactivity and hypoactivity of the system [84]. Chronic hyperactivity of the HPA axis has been reported in around 50% of MS patients, accompanied by high levels of cortisol in the CSF and blood [84]. Although it is thought that these changes are a reaction to persistent neuroinflammation and neurodegeneration, the precise connection between cortisol levels and MS pathology is still unclear. A flattened diurnal cortisol slope and higher evening cortisol levels are two aberrant cortisol patterns associated with depression in MS [85]. Relapsing–remitting MS patients are more likely to have greater anxiety and depression scores in addition to HPA axis hyperactivity, and these hormonal abnormalities are especially noticeable in these patients.

By regulating inflammatory cytokines including IL-1, IL-6, and prostaglandins, cortisol plays a crucial role in regulating immunological responses [86]. The HPA axis is stimulated by these cytokines, which increases the production of cortisol, which suppresses inflammation [86]. This regulating mechanism, however, is compromised in MS. According to studies, MS patients who have a hypoactive HPA axis typically see a more severe course of the disease, with more lesions and a greater degree of disability [87]. Conversely, patients with fewer lesions but more severe fatigue and mood disorders frequently exhibit hyperactivity of the HPA axis [87].

tDCS has shown potential in lowering cortisol levels [88] and preventing the cortisol level from rising [89], and this can be attributed to its impact on the HPA axis. One of the key ways tDCS lowers cortisol levels in MS is through its influence on the central regulation of the HPA axis. The HPA axis controls the release of cortisol in response to stress, and its dysregulation in MS patients often results in chronic hyperactivity and elevated cortisol, which exacerbates neuroinflammation and disease progression. tDCS, by targeting areas such as the prefrontal cortex, can modulate the HPA axis and restore its feedback control. This modulation reduces the excessive release of cortisol by improving the brain’s ability to regulate stress, thereby reducing neuroinflammation and potentially slowing disease progression.

tDCS may also lower cortisol by influencing the immune response in MS. We know that, in MS, pro-inflammatory cytokines such as IL-1 and IL-6 activate the HPA axis, leading to increased cortisol production. This is part of a feedback loop where cortisol acts to suppress inflammation. However, chronic HPA axis hyperactivity can result in desensitization to these cytokines, leading to ineffective immune suppression and ongoing inflammation. tDCS can disrupt this loop by reducing pro-inflammatory cytokine activity (this was detailed in another section), thereby decreasing the stimulus for cortisol production. This reduced inflammatory signaling lowers the burden on the HPA axis, contributing to a decrease in cortisol levels.

### 5.4. Cerebral Blood Flow Regulation

One of the human body’s most metabolically active organs is the brain. It requires a lot of energy. Brain tissue requires more than 20% of the body’s total oxygen [90]. However, its oxygen storage is constrained. Thus, sufficient cerebral blood flow (CBF) can supply the brain with oxygen, which is necessary for brain metabolism [91]. The intricate interplay of circulatory, respiratory, and brain physiology is necessary for the regulation of cerebral circulation. These physiological systems regulate hydrodynamic parameters (the cerebral vascular resistance and the arterial, intracranial, and venous pressures) in order to maintain a sufficient CBF in a healthy state [92].

Clinical measures suggest that a major feature of MS is vascular impairment [93]. In patients with relapsing–remitting or secondary progressive MS, cerebral blood flow is decreased in the grey [94] and white matter [95] regions of the brain. Given that individuals with the clinically isolated condition also have decreased cerebral blood flow, this may be an early illness characteristic [96]. Moreover, changes in CBF also occur in patients with depression [97]. According to Monkul et al. [98], a variety of CBF anomalies in the limbic–frontal-region resting-state blood flow are indicative of depressed patients. A lower CBF in the frontal lobe and anterior cingulate gyrus and a greater CBF in the temporal lobe, parietal lobe, thalamus, hippocampus, and posterior cingulate gyrus were the characteristics of depression [99]. With the exception of white matter in the bilateral posterior cerebral artery region and the right middle cerebral artery region, CBF in both the gray and white matter of the cerebral hemispheres was lower in patients with depressive disorders when compared to the control group [100].

tDCS increases regional CBF in MS patients [101] and in healthy individuals [102]. This effect occurs during stimulation and also after [102]. This effect may have beneficial effects on the overall pathophysiology of MS, and also on the depression that accompanies MS. Abnormal CBF in depression may result from endothelial dysfunction [103], which limits vasodilation, and tDCS might improve endothelial function, thus enhancing blood flow. Additionally, microglial overactivation refers to the excessive activation of microglial cells, the primary immune cells in the central nervous system, which play a key role in neuroinflammation. Overactive microglia release pro-inflammatory cytokines including IL-1β, IL-6, and TNF-α, which contribute to the disruption of cerebral circulation by impairing vascular function and promoting a pro-inflammatory environment [97]. Microglial overactivation can be reduced by tDCS [82], potentially decreasing inflammation and promoting better CBF. tDCS also modulates the hyperactivity of the HPA axis [88], which leads to elevated cortisol levels that impair vasodilation [97]. The modulation of the HPA axis and reduction in cortisol by tDCS could mitigate its negative impact on cerebral blood vessels, promoting improved CBF. Moreover, tDCS may influence CBF in depression by directly impacting neurovascular coupling and neurotransmitter regulation. Depression is characterized by imbalances in monoamine neurotransmitters like serotonin, norepinephrine, and dopamine (DA), which play a crucial role in maintaining vascular tone and CBF. For instance, DA, which is often reduced in depression, is known to protect cerebral autoregulation by modulating vascular responses to metabolic demands [97]. The increasing of dopamine activity by tDCS [104,105,106] might help restore proper cerebrovascular function, improving blood flow to areas like the prefrontal cortex, which is often hypoperfused in depression. Furthermore, tDCS may enhance the brain’s ability to regulate CBF by influencing the cortical excitability of regions like the DLPFC, a critical area involved in mood regulation. Increased excitability in this region could stimulate increased metabolic demand, leading to greater blood flow through the process of neurovascular coupling, where the increased neuronal activity drives an enhanced vascular response. This mechanism could be particularly important in reversing the reduced CBF observed in the frontal lobe and anterior cingulate cortex of depressed patients.

### 5.5. Increasing the Excitability of the Motor Cortex As an Additional Goal in Treating Depression in MS

Although the stimulation of the left DLPFC is the target of tDCS in treating depression, another possible additional target may be the stimulation of the left primary prefrontal cortex (M1). tDCS in MS is mainly used at two stimulation sites—the left DLPFC and the left M1. There is evidence of an asymmetry of motor cortex excitability in mood disorders, including depression [107]. A significant asymmetry was observed in major depressive disorder between motor threshold, a measure of cortical excitability thought to reflect neuronal membrane excitability [108], and intracortical facilitation, which may reflect excitatory inputs from glutamatergic pathways [109,110] and/or the depression of GABAergic function [111]. MT and ICF were found to be higher in the left motor cortex compared to the right, indicating the lower excitability of the left side compared to the right [109,110,112]. tDCS increases the excitability of the motor cortex, as first demonstrated in the work of Nitsche and Paulus [31]. This modulation is dependent on gamma-aminobutyric acid (GABA) and glutamate. Anodal tDCS applied to the primary motor cortex leads to a marked reduction in GABA levels in the targeted area, as detected by magnetic resonance spectroscopy (MRS), suggesting that this effect is partly driven by a decrease in GABAergic inhibition [113]. Conversely, cathodal tDCS results in a significant drop in glutamate, accompanied by a somewhat unexpected simultaneous decrease in GABA, implying that this reflects a reduction in excitatory glutamatergic activity [114]. Changes in GABA concentrations have been observed not only in the stimulated motor cortex but also in the functionally connected contralateral motor cortex, indicating that neurochemical alterations can occur beyond the directly stimulated area during plasticity processes [115]. To date, no studies have directly tested the effect of M1 stimulation on depression, but a notable large clinical trial in depression found that measuring the motor cortical excitability before treatment could help predict how well patients respond to tDCS. Specifically, a lower intracortical inhibition (reflecting a higher GABA-mediated inhibition) at baseline was linked to less improvement in depression symptoms when using anodal stimulation on the left and cathodal stimulation on the right dorsolateral prefrontal cortex [116]. This finding is particularly intriguing as it suggests that motor cortical excitability could serve as a biomarker for predicting antidepressant effectiveness, shedding light on the motor cortex’s role in both depression and treatment outcomes [117]. It is important to note that Cotovio et al. [107] emphasize that the reduced excitability of the left M1 in depression does not only mean that the left M1 is a potential target for stimulation, which rather indicates a general cortical and hemispheric asymmetry in depression, amounting to the stimulation of the left hemisphere in mood disorders.

## 6. Limitations and Future Directions

This mechanistic review found little or no results in the areas it covered. It is, therefore, important to highlight the limitations of the research to date to facilitate further work.

### 6.1. Sample Size and Generalizability

The statistical power of the results is severely limited by the small sample sizes employed in the included studies, which has an impact on the capacity to identify significant benefits of tDCS on depression and anxiety in MS patients. Less than 20 individuals were included in the majority of the studies, with some having as low as 11 or 6 patients, which raises the possibility of type II errors (failure to identify a real effect). Furthermore, it is challenging to extrapolate the findings from small samples to the larger MS community, which varies widely in terms of comorbidities, symptom severity, and disease progression. Applying these findings to a larger group of MS patients is made more difficult by the heterogeneity in patient variables, such as the age, length of disease, and baseline neuropsychiatric symptom levels. To establish the genuine effectiveness of tDCS in treating depression and anxiety in MS patients and to guarantee that the findings can be safely implemented in clinical practice, larger adequately powered trials are required.

### 6.2. More Studies Examining the Possible Mechanisms of Action of tDCS

The findings that tDCS works in a given disorder are already too few in contemporary neuromodulation research. Studies examining the mechanisms of tDCS, preferably at the brain level, are very much needed and valuable. Regarding the publications included in this review, only one study used electrophysiological measurements to examine the mechanisms (frontal–midline theta). Future studies using EEQ, QEEG, and, above all, fMRI are necessary to discover how tDCS works on depression and anxiety in MS. It is necessary to verify whether tDCS affects the altered connectivity and activity of the brain, or whether it affects different brain networks important for the pathophysiology of MS. It is known that tDCS induces changes in brain activity visible in fMRI [118] and acts on brain networks [119]. Similarly, tDCS induces changes in EEG (QEEG) in various pathophysiological states [120]. In future studies, it is important not to limit the analysis to single EEG parameters, such as absolute or relative spectral power, but also to include others, such as evoked potentials. Moreover, in addition to EEG in the resting state, it is also possible to examine EEG in various cognitive states and using various paradigms.

In addition, it is worth checking the effect of tDCS on various biomarkers from the blood, such as cytokines or cortisol. This type of diagnostics is also valuable, and is much cheaper than neuroimaging methods. Several hypotheses can be proposed based on studies from other diseases regarding the effects tDCS may induce in patients with neuropsychiatric dysfunctions in MS. Regarding the effect on neuroinflammation, it is important to confirm whether reducing microglial activity by tDCS could decrease the inflammatory response, protect against further neurodegeneration, and induce neuroprotective effects that mitigate both MS progression and depressive symptoms. Another hypothesis concerns the regulation of the HPA axis. It is important to verify whether tDCS is able to reduce the activity of the HPA axis by reducing cortisol levels. Future studies should include measurements of morning cortisol levels in saliva, blood, or both.

### 6.3. Baseline Symptom Severity

This study’s major drawback is the large number of studies that included MS patients who did not show appreciable baseline levels of anxiety or depression. Pre-treatment scores on standardized depression and anxiety scales (e.g., HADS or BDI) were below the clinical thresholds in multiple trials, suggesting that participants were either asymptomatic or exhibited minor symptoms at the beginning of the condition. Given that it is difficult to show that tDCS is successful in treating depression or anxiety when these symptoms are absent or just mildly expressed, it is likely that the absence of significant baseline symptoms led to the lack of meaningful post-treatment improvements. To better assess the effectiveness of tDCS in treating these symptoms in the MS population, future research should concentrate on enrolling patients with moderate to severe baseline levels of anxiety and depression.

### 6.4. Variability in Study Designs

The next major constraint in assessing the overall efficacy of tDCS for treating depression and anxiety in MS patients is the heterogeneity of study designs among the included research. The comparison of results is complicated by important variations in stimulation parameters, including the current intensity (1 mA to 2 mA), the number of sessions (5 to 10), and the duration of each session (20 min on average, although varied in other studies). Furthermore, different works of research used different electrode placements; some targeted the motor cortex and others the posterior parietal cortex, or the dorsolateral prefrontal cortex. Since different brain regions may be more or less successful at modulating symptoms of anxiety and depression, these variations in electrode location probably had an impact on the results. Additionally, some studies employed different stimulation protocols (e.g., continuous versus intermittent stimulation), which could impact the efficacy of tDCS. This lack of standardization makes it difficult to determine the most effective tDCS protocol for MS patients and limits the ability to draw consistent conclusions about the intervention’s therapeutic potential. To improve the comparability and reliability of the results, future studies should adopt standardized tDCS protocols, focusing on consistent parameters such as current intensity, session frequency, and electrode placement.

### 6.5. Short Treatment Duration

The next important restriction that could have contributed to the lack of significant improvements in depression and anxiety is the short treatment duration in the majority of the included studies. Most of the research used tDCS treatments that lasted only five sessions over the course of a week, which could not have been stimulating enough to yield significant or long-lasting therapeutic effects. Studies on the use of tDCS for different illnesses, like major depressive disorder, indicate that it usually takes at least 10 sessions to see clinically meaningful changes. Some of these studies even advise lengthier treatment regimens for long-term benefits. The capacity to identify any significant changes was probably hampered by the short duration of the protocols in this review, especially for individuals with mild or moderate symptoms. Furthermore, it is thought that tDCS has cumulative effects, and brief treatment intervals might not provide these effects enough time to become apparent. To completely evaluate the potential effectiveness of tDCS in treating depression and anxiety in MS patients, future research should examine the effects of extended treatment durations, with more sessions spaced out over many weeks. We recommend that treatment protocols include at least 10 tDCS sessions, with one session per day for a 2-week period. Currently we do not have data on the optimal current intensity. However, the two most commonly used current intensities in adults should be considered: 1.5 mA and 2 mA. Repeated stimulation protocols may also be necessary, given the progressive nature of MS. Additionally, in 24 h care settings, such as hospitals or rehabilitation centers, the efficacy of stimulation performed twice daily at 10 h intervals should be evaluated. During stimulation, cognitive training can be performed simultaneously, as it is known that people with MS have a decline in cognitive functions [121]. The advantage of tDCS is that it can be used together with other interventions—including those performed at the same time as tDCS. Research shows that tDCS combined with cognitive training (CT) is effective in improving working memory in healthy older people [122], and in improving cognitive functions in Alzheimer’s disease [123] and neuropsychiatric disorders [124]. tDCS appears to amplify the benefits of CT by helping users make the most of it. Combining tDCS with CT not only fosters new connections between brain cells but also sparks substantial excitatory changes in the cerebral cortex. This combination enhances neuroplasticity, supports memory consolidation, and, ultimately, boosts cognitive performance [125,126,127]. Additionally, repeated exposure to stimulation—especially 10 times or more—can create a cumulative, synergistic effect that progressively improves brain function [128]. After each session, it may take time for neuronal connections to strengthen and the neural network to optimize, with repeated sessions leading to more pronounced benefits over time [129,130]. In order to ascertain whether the benefits of tDCS persist over time, longer follow-up times would also be advantageous.

### 6.6. Lack of Standardized Outcome Measures

Another important drawback that makes it difficult to compare results and make consistent conclusions regarding how well tDCS treats depression and anxiety in MS patients is the lack of defined end measures in all of the included trials. A variety of instruments, including the Hospital Anxiety and Depression Scale (HADS), the Beck Depression Inventory (BDI), the Visual Analogue Scale (VAS), and the Depression, Anxiety, and Stress Scale (DASS), were used in different studies to measure anxiety and depression. Although these methods are legitimate in and of themselves, it is challenging to directly evaluate the magnitude of changes between studies due to the differences in how these tools quantify symptoms. Additionally, some research only included graphical data without exact numerical values, which made it harder to comprehend the findings. Inconsistencies in outcomes were also a result of using alternative measuring time points, such as follow-up assessments versus assessments just after therapy. This lack of consistency restricts the generalizability of the conclusions about the efficacy of tDCS and makes it more difficult to combine findings across trials. Future studies should standardize the use of validated outcome measures to promote comparability. This will ensure that anxiety and depression are measured consistently and on the same scales, facilitating more trustworthy cross-study comparisons and meta-analyses.

## 7. Conclusions

This mechanistic review examined the efficacy of tDCS in reducing neuropsychiatric symptoms such as depression and anxiety in MS and found no statistically significant effects in most of the included studies. This may be partly due to the fact that many participants in these studies did not have pre-treatment scores indicative of depression or anxiety disorders, which limited the ability to observe measurable improvements. In the few studies where patients had elevated baseline scores, some improvement in post-treatment scores was observed, although these findings are limited by small sample sizes and inconsistent methodologies. The included studies also have several limitations, such as a small number of participants, few stimulation sessions, and a lack of patients with clinically diagnosed neuropsychiatric conditions. Despite these limitations, evidence from studies in other populations suggests that tDCS may hold promise in reducing symptoms of depression and anxiety. However, its efficacy in treating neuropsychiatric symptoms in MS remains unproven, and further well-designed studies are needed to establish its therapeutic potential.

## Figures and Tables

**Table 1:** Studies included in the review.

Post-Treatment Anxiety Outcome	Post-Treatment Depression Outcome	Pre-Treatment Anxiety Score	Pre-Treatment Depression Score	tDCS Target Area (Anode)	Number of Sessions, Session Duration	Current Intensity (mA)	Patients (*n*)	Study References
Significant decrease (HADS: ~3.6, no anxiety)	No significant decrease (HADS: ~6, no depression)	HADS: ~5.6 (no anxiety)	HADS: ~7 (no depression)	Left DLPFC (F3)	5, 20 min	2	11	[43]
Significant decrease in PPC group (HADS: 2.0, no anxiety)	Significant decrease in PPC group (HADS: 1.9, no depression)	HADS anxiety score: DLPFC group: 4.6, PPC group: 5.7 (both indicate no anxiety)	HADS depression score: DLPFC group: 3.9, PPC group: 4.2 (both indicate no depression)	DLPFC and PPC	5, 20 min	2	10	[44]
No significant decrease (lack of numerical data	No significant decrease (lack of numerical data)	HADS anxiety score: 4	BDI: 4, HADS depression score: 2	Left DLPFC (F3)	5, 20 min	1	13	[45]
Non-significant decrease in VAS (~30, moderate anxiety)	Non-significant decrease (BDI: ~7, minimal depression)	VAS: ~40 (moderate anxiety)	BDI: ~11 (minimal depression)	C3 or C4	5, 20 min	2	19, active tDCS = 10, sham tDCS = 9	[46]
Non-significant decrease (HADS: 7.6, normal anxiety)	Non-significant decrease (HADS: 6.0, normal depression)	HADS: 7.7 (normal anxiety)	HADS: 6.4 (normal depression)	Left DLPFC (F3)	6, 20	2	16	[47]
Non-significant decrease (DASS: 7.1, no anxiety)	Non-significant decrease (DASS: 6.6, no depression)	DASS: 7.9 (no anxiety)	DASS: 6.9 (no depression)	C3 or C4	5, 20	2	30, active tDCS = 15, sham tDCS = 15	[48]
Not measured	Non-significant decrease (BDI: 11.5, minimal depression)	Not measured	BDI: 11.7 (minimal depression)	M1	5, 20	2	6	[49]
Not measured	Significant decrease (HDRS: 11, mild depression)	Not measured	HDRS: 15 (mild depression)	Left DLPFC (F3)	40, 20 min	2	1	[50]
Not measured	No significant improvement (BDI: ~12, no depression)	Not measured	BDI: ~12 (no depression)	Somatosensory cortex (C3 or C4)	5, 20 min	2	20, active tDCS = 10, sham tDCS = 10	[51]
Not measured	Significant improvement (BDI: 10.58, minimal depression)	Not measured	BDI: 13.83 (minimal-mild depression)	Left DLPFC (F3)	10, 20 min	2	12	[52]
Significant decrease (DASS: 1.60, no anxiety)	Significant decrease (DASS: 2.06, no depression)	DASS: 3.00 (no anxiety)	DASS: 3.73 (no depression)	Left DLPFC (F3)	10, 20 min	1.5	37, active tDCS = 19, sham tDCS = 18	[53]

Abbreviations: HADS—Hospital Anxiety and Depression Scale, BDI—Beck Depression Inventory, VAS—Visual Analogue Scale, DASS—Depression, Anxiety, and Stress Scale, HDRS—Hamilton Depression Rating Scale.

**Table 2:** Characteristics of the patients included in the studies.

Medications	Symptom History (Months or Years)	Severity	Mental Health Condition	Duration of Illness (in Months or Years)	Handedness	Type of MS	Gender (M/F)	Mean Age (Years)	Sample Size	Study
Immunomodulatory treatment (9 patients)	66.36 ± 47.45 months (7–137)	Moderate to severe	Fatigue, anxiety	75.64 ± 45.97 months (17–136)	No data	10 patients had relapsing–remitting MS and 1 patient had a secondary progressive course	3/8	43.91 ± 9.69, range: 26–57 years	11	[43]
Dimethyl fumarate (*n* = 6), natalizumab (*n* = 3), or monthly intravenous methylprednisolone (*n* = 1)	No data	Moderate	Fatigue	14.0 ± 9.9 years	No data	9 patients had relapsing–remitting MS and 1 patient had a secondary progressive course	6/4	40.50 ± 11.18, range: 26–63 years	10	[44]
Interferon beta (*n* = 3), glatiramer acetate (*n* = 4), or natalizumab (*n* = 3)	No data	Varied	Fatigue	9.0 years (all patients), 9.4 years (responders), and 8.5 years (non-responders)	Only right-handed patients	Only relapsing–remitting	3/10	46.8 ± 6.8, range: 37–59	13	[45]
In tDCS group: gabapentin, pregabalin, carbamazepine, levetiracetam, oxcarbazepine, paracetamole, and amitriptiline	2.79 years	Severe	Chronic neuropathic pain	10.21 years	No data	Relapsing–remitting	8/11	44.8 ± 27.5	19	[46]
Antiepileptic drugs (nine patients), antidepressants (nine patients), opioids (five patients), and immunomodulating agents (13 patients)	More than 3 months	Severe	Chronic neuropathic pain	11.8 ± 9.4 years	Only right-handed patients	11 patients had relapsing–remitting MS, 4 were in the secondary progressive phase and one had a primary progressive type	3/13	48.9 ± 10.0, range: 38–67	16	[47]
Antidepressants, pain medications	No data	No data	Chronic neuropathic pain	Real tDCS group: 1–5 years: 2 patients, 6–10 years: 1 patient, and more than 10 years: 12 patients	No data	Real tDCS group: relapsing–remitting (*n* = 9), primary progressive (*n* = 1), and secondary progressive (*n* = 4)	Real tDCS group: 4/11	Real tDCS group: 51.2	30	[48]
No data	No data	Moderate	Fatigue, depression, pain	No data	No data	Relapsing–remitting	3/3	46.7 ± 14.1	6	[49]
No data	No data	Severe	Depression, cognitive impairment	20 years	No data	Secondary progressive	1 female	54	1	[50]
No specific medication intervention	No data	Moderate	Sensory deficit	Real tDCS group: 5.1 years	No data	Relapsing–remitting	8/12	25–61 years	20	[51]
Exercise and tDCS were combined, no medications specified	No data	Moderate	Depression, fatigue	16.65 ± 7.44	No data	Relapsing–remitting (*n* = 7), and secondary progressive (*n* = 5)	9/6 (before exclusion)	48.08 ± 8.55	12	[52]
Immunosuppressant (*n* = 17)	No data	Varied severity of cognitive and emotional deficits	Sleep difficulties, or psychological distress	Real tDCS group: 1–10 years (*n* = 14), 11–20 years (*n* = 4), and more than 20 years (*n* = 1)	No data	Real tDCS group: relapsing–remitting (*n* = 15), clinically isolated syndrome (*n* = 3), and secondary progressive (*n* = 2)	Real tDCS group: 4/15	Real tDCS group: 36.89	37	[53]

**Table 3:** Risk of bias assessment in included studies.

Overall Bias Risk	Selective Reporting	Incomplete Outcome Data	Blinding of Participants/Personnel	Allocation Concealment	Random Sequence Generation	Study
Moderate	High risk (graph only)	Low risk	Low risk	Low risk	Low risk	[43]
Moderate	High risk (graph only)	Low risk	Low risk	Low risk	Low risk	[44]
Moderate	High risk (graph only)	Low risk	Low risk	Low risk	Low risk	[45]
Moderate	High risk (graph only)	Low risk	Low risk	Low risk	Low risk	[46]
Low	Low risk	Low risk	Low risk	Low risk	Low risk	[47]
Moderate	Low risk	Low risk	Single-blind	Unclear	Low risk	[48]
Moderate	High risk (small sample)	Low risk	Low risk	Low risk	Low risk	[49]
High (case study)	Low risk	Low risk	Not blinded	Not applicable	Not applicable	[50]
Low	Low risk	Low risk	Low risk	Low risk	Low risk	[51]
High	Low risk	Low risk	High risk (not blinded)	Not applicable	High (not randomized)	[52]
Low	Low risk	Low risk	Low risk	Low risk	Low risk	[53]

## Data Availability

No new data were created or analyzed in this study. Data sharing is not applicable to this article.
